# Chromosome-Scale Assembly of the Complete Genome Sequence of Leishmania (Mundinia) orientalis, Isolate LSCM4, Strain LV768

**DOI:** 10.1128/MRA.00574-21

**Published:** 2021-09-09

**Authors:** Hatim Almutairi, Michael D. Urbaniak, Michelle D. Bates, Narissara Jariyapan, Waleed S. Al-Salem, Rod J. Dillon, Paul A. Bates, Derek Gatherer

**Affiliations:** a Division of Biomedical & Life Sciences, Faculty of Health & Medicine, Lancaster University, Lancaster, United Kingdom; b Ministry of Health, Riyadh, Saudi Arabia; c Department of Parasitology, Faculty of Medicine, Chulalongkorn University, Bangkok, Thailand; Vanderbilt University

## Abstract

Leishmania (Mundinia) orientalis is a kinetoplastid parasite first isolated in 2014 in Thailand. We report the complete genome sequence of *L.* (*M.*) *orientalis*, sequenced using combined short-read and long-read technologies. This will facilitate greater understanding of this novel pathogen and its relationship to other members of the subgenus *Mundinia*.

## ANNOUNCEMENT

Leishmaniasis is spread through sand fly bites and caused by kinetoplastid parasites of the genus *Leishmania* (1). It is present in over 90 countries, infecting approximately 12 million people and putting 350 million more at risk of infection from visceral, cutaneous, or mucocutaneous leishmaniasis (2, 3). The genus *Leishmania* is subdivided into four subgenera, *Leishmania*, *Sauroleishmania*, *Viannia*, and most recently *Mundinia* (4, 5), the latter being the least studied. *Mundinia* includes a wide range of species with different hosts and regional distributions (6), including Thailand, where leishmaniasis is an emerging disease (7–9). Leishmania orientalis was formally described as part of *Mundinia* in 2018 (10). We report here the complete genome sequence of Leishmania (Mundinia) orientalis, isolate LSCM4, strain LV768 (WHO code MHOM/TH/2014/LSCM4), originally obtained from a cutaneous biopsy specimen from a 57-year-old woman from northern Thailand (10).

Parasites were grown using an *in vitro* culture system previously developed for *L.* (*M.*) *orientalis* axenic amastigotes (11), in Schneider’s insect medium at 26°C as promastigotes, then in M199 medium supplemented with 10% fetal calf serum (FCS), 2% stable human urine, 1% basal medium Eagle vitamins, and 25 μg/ml gentamicin sulfate, with subpassage to fresh medium every 4 days to sustain the parasite growth and viability. DNA was extracted and purified using a Qiagen DNeasy blood and tissue kit using the spin column protocol, according to the manufacturer’s instructions. The extracted DNA concentration was assessed using a Qubit fluorometer, microplate reader, and agarose gel electrophoresis. All sequencing libraries were based on the same extracted DNA sample to avoid any inconsistency.

Short-read library construction and sequencing were contracted to (i) BGI (Shenzhen, China) for DNBSEQ libraries, producing paired-end reads (170 bp, 270 bp, and 500 bp) using the Illumina HiSeq platform, and (ii) Aberystwyth University (Aberystwyth, UK) for TruSeq Nano DNA libraries, producing paired-end reads (300 bp) using the Illumina MiSeq platform. We performed long-read library preparation and sequencing according to the Nanopore protocol (SQK-LSK109) on R9 flow cells (FLO-MIN106). The read quality was assessed using MultiQC (12), incorporating the use of FastQC for Illumina short reads and pycoQC for Nanopore long reads.

We assembled the long reads using Flye (13), with default parameters, to generate chromosome-scale scaffolds. Then, using Minimap2 (14) and SAMtools (15), we mapped the short reads onto the assembled scaffolds to correct erroneous bases within the long reads and create consensus sequences. After polishing the assembly with Pilon (16), another round of consensus short-read mapping was performed. Then, we removed the duplicated contigs and sorted the remainder according to length using Funannotate (17, 18). Finally, we separated the chimeric sequences and performed scaffolding using RaGOO (19) with the Leishmania major Friedlin strain genome (GenBank accession number GCA_000002725.2) (20) as a reference guide, aligning all 36 chromosomes for our assembly, thereby also determining the chromosome ends to be complete, with the exception of 62 small contigs totaling 257,579 bp.

**FIG 1:**
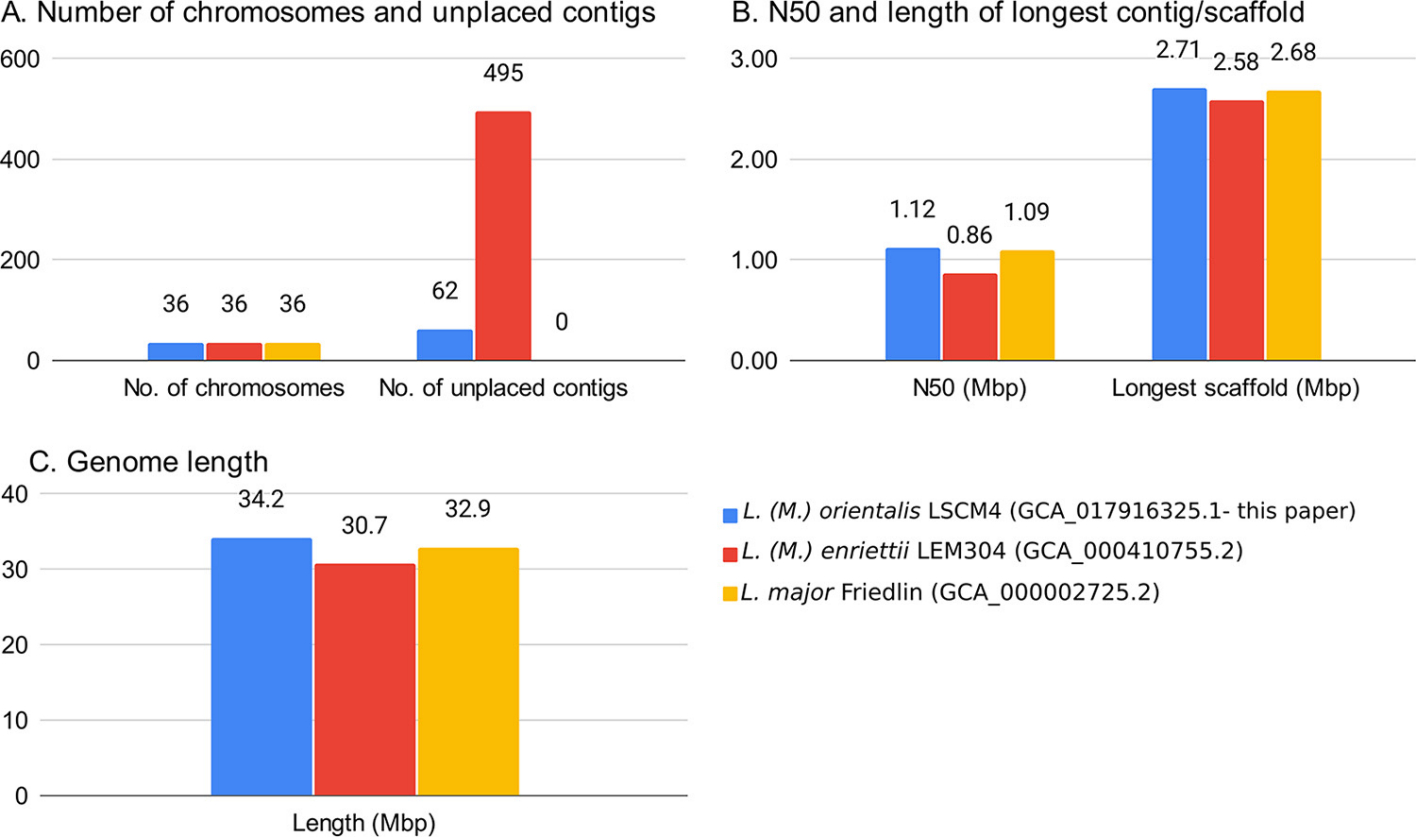
Assembly comparison of *L.* (*M.*) *orientalis* LV768 with *L.* (*M.*) *enriettii* LEM3045 and L. major Friedlin.

The analysis workflow for assembly, repeat masking, and annotation was performed using Snakemake (21); it is available online for reproducibility purposes (https://github.com/hatimalmutairi/LGAAP), including the software versions and parameters used (22). Figure 1 compares our assembly with other complete genomes.

We assessed the assembly completeness using BUSCO (23), with the lineage data set for the phylum *Euglenozoa*, containing 130 single-copy orthologs from 31 species, and we found 128 of the orthologs to be present (98.5% completeness). We carried out functional annotation and prediction using the MAKER2 (24) annotation pipeline in combination with AUGUSTUS (25) gene prediction software, with the predictor trained on Leishmania tarentolae. Table 1 shows additional summary metrics for the sequencing, assembly, and annotation.

**TABLE 1:** Detailed summary metrics of the genome sequencing, assembly, and annotation for *L.* (*M.*) *orientalis* LV768

Feature(s)	Metric(s)
Total no. of reads	80,540,904
No. of MiSeq reads	3,831,060
No. of HiSeq reads	76,124,074
No. of MinION reads (read *N*_50_ [bp])	585,770 (11,497)
No. of bases (Gb)	29.20
Genome coverage (×)	390.7
Total no. of scaffolds	98
Genome size (bp)	34,194,276
*N*_50_ (bp)	1,120,138
GC content (%)	59.70
No. of Ns (% of genome)	1,707 (0.005)
No. of genes	8,158
Gene density (no. of genes/Mb)	238.6
No. of exons	8,488
Mean gene length (bp)	1,938
Total length of CDSs^*a*^ (Mb) (% of genome)	15.40 (45.05)

a CDSs, coding DNA sequences.

### Data availability.

The assembly and annotations are available under GenBank assembly accession number GCA_017916335.1. The master record for the whole-genome sequencing project is available under accession number JAFHLR000000000.1. The raw sequence reads are available at PRJNA691532.

## References

[1] Burza S, Croft SL, Boelaert M. 2018. Leishmaniasis. Lancet 392:951–970. doi:10.1016/S0140-6736(18)31204-2. 30126638

[2] Ikeogu NM, Akaluka GN, Edechi CA, Salako ES, Onyilagha C, Barazandeh AF, Uzonna JE. 2020. *Leishmania* immunity: advancing immunotherapy and vaccine development. Microorganisms 8:1201. doi:10.3390/microorganisms8081201. PMC7465679 32784615

[3] Alvar J, Velez ID, Bern C, Herrero M, Desjeux P, Cano J, Jannin J, den Boer M, WHO Leishmaniasis Control Team. 2012. Leishmaniasis worldwide and global estimates of its incidence. PLoS One 7:e35671. doi:10.1371/journal.pone.0035671. 22693548 PMC3365071

[4] Muniz J, Medina H. 1948. Cutaneous leishmaniasis of the guinea pig, *Leishmania enriettii* n. sp. Hospital (Rio J) 33:7–25. 18908199

[5] Espinosa OA, Serrano MG, Camargo EP, Teixeira MMG, Shaw JJ. 2018. An appraisal of the taxonomy and nomenclature of trypanosomatids presently classified as *Leishmania* and *Endotrypanum*. Parasitology 145:430–442. doi:10.1017/S0031182016002092. 27976601

[6] Sereno D. 2019. *Leishmania* (*Mundinia*) spp.: from description to emergence as new human and animal *Leishmania* pathogens. New Microbes New Infect 30:100540. doi:10.1016/j.nmni.2019.100540. 31061710 PMC6487459

[7] Pothirat T, Tantiworawit A, Chaiwarith R, Jariyapan N, Wannasan A, Siriyasatien P, Supparatpinyo K, Bates MD, Kwakye-Nuako G, Bates PA. 2014. First isolation of *Leishmania* from Northern Thailand: case report, identification as *Leishmania martiniquensis* and phylogenetic position within the *Leishmania enriettii* complex. PLoS Negl Trop Dis 8:e3339. doi:10.1371/journal.pntd.0003339. 25474647 PMC4256172

[8] Thisyakorn U, Jongwutiwes S, Vanichsetakul P, Lertsapcharoen P. 1999. Visceral leishmaniasis: the first indigenous case report in Thailand. Trans R Soc Trop Med Hyg 93:23–24. doi:10.1016/s0035-9203(99)90166-9. 10492782

[9] Maharom P, Siripattanapipong S, Mungthin M, Naaglor T, Sukkawee R, Pudkorn R, Wattana W, Wanachiwanawin D, Areechokchai D, Leelayoova S. 2008. Visceral leishmaniasis caused by *Leishmania infantum* in Thailand. Southeast Asian J Trop Med Public Health 39:988–990. 19062685

[10] Jariyapan N, Daroontum T, Jaiwong K, Chanmol W, Intakhan N, Sor-Suwan S, Siriyasatien P, Somboon P, Bates MD, Bates PA. 2018. *Leishmania* (*Mundinia*) *orientalis* n. sp. (Trypanosomatidae), a parasite from Thailand responsible for localised cutaneous leishmaniasis. Parasit Vectors 11:351. doi:10.1186/s13071-018-2908-3. 29914526 PMC6006788

[11] Chanmol W, Jariyapan N, Somboon P, Bates MD, Bates PA. 2019. Axenic amastigote cultivation and *in vitro* development of *Leishmania orientalis*. Parasitol Res 118:1885–1897. doi:10.1007/s00436-019-06311-z. 30972571

[12] Ewels P, Magnusson M, Lundin S, Kaller M. 2016. MultiQC: summarize analysis results for multiple tools and samples in a single report. Bioinformatics 32:3047–3048. doi:10.1093/bioinformatics/btw354. 27312411 PMC5039924

[13] Kolmogorov M, Yuan J, Lin Y, Pevzner PA. 2019. Assembly of long, error-prone reads using repeat graphs. Nat Biotechnol 37:540–546. doi:10.1038/s41587-019-0072-8. 30936562

[14] Li H. 2016. Minimap and miniasm: fast mapping and *de novo* assembly for noisy long sequences. Bioinformatics 32:2103–2110. doi:10.1093/bioinformatics/btw152. 27153593 PMC4937194

[15] Li H, Handsaker B, Wysoker A, Fennell T, Ruan J, Homer N, Marth G, Abecasis G, Durbin R, 1000 Genome Project Data Processing Subgroup. 2009. The Sequence Alignment/Map format and SAMtools. Bioinformatics 25:2078–2079. doi:10.1093/bioinformatics/btp352. 19505943 PMC2723002

[16] Walker BJ, Abeel T, Shea T, Priest M, Abouelliel A, Sakthikumar S, Cuomo CA, Zeng Q, Wortman J, Young SK, Earl AM. 2014. Pilon: an integrated tool for comprehensive microbial variant detection and genome assembly improvement. PLoS One 9:e112963. doi:10.1371/journal.pone.0112963. 25409509 PMC4237348

[17] Li W-C, Wang T-F. 2021. PacBio long-read sequencing, assembly, and Funannotate reannotation of the complete genome of *Trichoderma reesei* QM6a. Methods Mol Biol 2234:311–329. doi:10.1007/978-1-0716-1048-0_21. 33165795

[18] Palmer JM, Stajich J. 2020. Funannotate v1.8.1: eukaryotic genome annotation. doi:10.5281/zenodo.1134477.

[19] Alonge M, Soyk S, Ramakrishnan S, Wang X, Goodwin S, Sedlazeck FJ, Lippman ZB, Schatz MC. 2019. RaGOO: fast and accurate reference-guided scaffolding of draft genomes. Genome Biol 20:224. doi:10.1186/s13059-019-1829-6. 31661016 PMC6816165

[20] Ivens AC, Peacock CS, Worthey EA, Murphy L, Aggarwal G, Berriman M, Sisk E, Rajandream M-A, Adlem E, Aert R, Anupama A, Apostolou Z, Attipoe P, Bason N, Bauser C, Beck A, Beverley SM, Bianchettin G, Borzym K, Bothe G, Bruschi CV, Collins M, Cadag E, Ciarloni L, Clayton C, Coulson RMR, Cronin A, Cruz AK, Davies RM, De Gaudenzi J, Dobson DE, Duesterhoeft A, Fazelina G, Fosker N, Frasch AC, Fraser A, Fuchs M, Gabel C, Goble A, Goffeau A, Harris D, Hertz-Fowler C, Hilbert H, Horn D, Huang Y, Klages S, Knights A, Kube M, Larke N, Litvin L, et al. 2005. The genome of the kinetoplastid parasite, *Leishmania major*. Science 309:436–442. doi:10.1126/science.1112680. 16020728 PMC1470643

[21] Mölder F, Jablonski KP, Letcher B, Hall MB, Tomkins-Tinch CH, Sochat V, Forster J, Lee S, Twardziok SO, Kanitz A, Wilm A, Holtgrewe M, Rahmann S, Nahnsen S, Köster J. 2021. Sustainable data analysis with Snakemake. F1000Res 10:33. doi:10.12688/f1000research.29032.2. 34035898 PMC8114187

[22] Almutairi H, Urbaniak MD, Bates MD, Jariyapan N, Kwakye-Nuako G, Thomaz-Soccol V, Al-Salem WS, Dillon RJ, Bates PA, Gatherer D. 2021. LGAAP: *Leishmaniinae* genome assembly and annotation pipeline. Microbiol Resour Announc 10:e00439-21. doi:10.1128/MRA.00439-21. PMC8297458 34292068

[23] Simao FA, Waterhouse RM, Ioannidis P, Kriventseva EV, Zdobnov EM. 2015. BUSCO: assessing genome assembly and annotation completeness with single-copy orthologs. Bioinformatics 31:3210–3212. doi:10.1093/bioinformatics/btv351. 26059717

[24] Holt C, Yandell M. 2011. MAKER2: an annotation pipeline and genome-database management tool for second-generation genome projects. BMC Bioinformatics 12:491. doi:10.1186/1471-2105-12-491. 22192575 PMC3280279

[25] Stanke M, Steinkamp R, Waack S, Morgenstern B. 2004. AUGUSTUS: a Web server for gene finding in eukaryotes. Nucleic Acids Res 32:W309–W312. doi:10.1093/nar/gkh379. 15215400 PMC441517

